# Genetic contexts related to the diffusion of plasmid-mediated CTX-M-55 extended-spectrum beta-lactamase isolated from *Enterobacteriaceae* in China

**DOI:** 10.1186/s12941-018-0265-x

**Published:** 2018-03-23

**Authors:** Xiaoxin Hu, Jianjun Gou, Xiaobing Guo, Zaiqiu Cao, Yuan Li, Hongjian Jiao, Xiaohong He, Yihui Ren, Fuyun Tian

**Affiliations:** 1 Department of Clinical Laboratory, First Affiliated Hospital of Zhengzhou University, Zhengzhou, Henan China; 2 Key Laboratory of Laboratory Medicine of Henan Province, Zhengzhou, Henan China; 3 School of Medicine, Pusan National University, Busan, Republic of Korea; 4 Department of Medical Laboratory Technology, Xinyang Vocational and Technical College, Xinyang, Henan China

**Keywords:** *bla*_CTX-M-55_, *Enterobacteriaceae*, ISEcp1

## Abstract

**Background:**

CTX-M-55 extended-spectrum beta-lactamases are being rapidly disseminated and transmitted in clinical practices around the world. The genetic contexts of the transferable plasmid-mediated *bla*_CTX-M-55_ gene in *Enterobacteriaceae* were detected and characterized in this study.

**Methods:**

Isolates were obtained from the First Affiliated Hospital of Zhengzhou University between September 2015 and March 2016. Based on polymerase chain reaction and BLAST analysis, resistance genes and genetic context of the *bla*_CTX-M-55_ gene were investigated. Conjugation experiments and multilocus sequence typing were performed to demonstrate plasmid-mediated *bla*_*CTX*-*M*-*55*_ transmission.

**Results:**

Thirteen *bla*_CTX-M-55_-positive isolates of *Enterobacteriaceae* were obtained. Seven isolates were *Escherichia coli*, 3 were *Klebsiella pneumoniae*, 1 was *Citrobacter freundii*, 1 was *Morganella morganii* and 1 was *Serratia marcescens*. The *bla*_CTX-M-55_ gene has not previously been identified from *C. freundii* and *M. morganii*. Four different *bla*_*CTX*-*M*-*55*_ genetic contexts were identified, and all of them harbored *ISEcp1* in the region upstream of *bla*_CTX-M-55_ (in two cases, *ISEcp1* was truncated by *IS26*, and in one case, it was truncated by *IS1294*), whereas *ORF477* was detected downstream of the *bla*_CTX-M-55_ gene from 12 of 13 strains. The novel genetic context of *ISEcp1∆*-*bla*_*CTX*-*M*-*55*_-*∆IS903* was firstly detected the *IS903* element which was identified downstream of *bla*_CTX-M-55_. A conjugation assay revealed that all *bla*_*CTX*-*M*-*55*_ plasmids were quickly and easily transferable to recipient *E. coli*, which then presented resistance to multiple antibiotics.

**Conclusions:**

Numerous *bla*_*CTX*-*M*-*55*_-positive strains were isolated in a short period of 7 months. The findings indicate that *bla*_*CTX*-*M*-*55*_ was rapidly disseminated. The genetic context and conjugative transfer found in this study demonstrate that there is active transmission of *bla*_CTX-M-55_ among strains of *Enterobacteriaceae* in China, which could give rise to an urgent global public health threat.

## Background

Since the first reports of CTX-M extended-spectrum beta-lactamases (ESBLs) in 1989 [1], at least 26 bacterial species across the world have been referenced in the “CTX-M pandemic” [2]. More than 190 diverse variants of CTX-M have been recorded to date. Among these variants, CTX-M-55 pertains to the CTX-M-1 cluster, which is a variant of CTX-M-15 with only one amino acid substitution (Ala-80-Val) [3]. This variant was first reported in 2006 [4] and was identified in Thailand as well as in the UK [3–5]. Over the past decade, the isolate rate of CTX-M-55 in *Escherichia coli* from animals has been increasingly raised. However, CTX-M-55 was not identified in clinical practices in China until 2010, when it was detected from a person who traveled to China [6]. Since then, plenty of surveys have confirmed the emergence of *bla*_*CTX*-*M*-*55*_ among clinical pathogenic in China [7–11].

Conjugative plasmids are one of the most important mechanisms for the appearance and spread of *bla*_*CTX*-*M*_. These plasmids facilitate horizontal transfer to other isolates and even cross-species barriers [12]. Insertion sequences (ISs), which cause insertion mutations and genome rearrangements, are the smallest mobile elements (< 2.5 Kb) independent transposition in an organism and competent to promote translocation, and the transferability of a resistance gene will largely increased under the mediated of ISs [13]. Various types of genetic platforms are associated with *bla*_*CTX*-*M*_ genes, and *ISEcp1* is frequently recorded upstream of *bla*_*CTX*-*M*_. *ISEcp1* can transpose the *bla*_*CTX*-*M*_ gene and act as a strong activator for the high expression of it [12, 14, 15]. In addition, other insertion sequences, including *IS26*, *IS903* and *ORF477*, are also frequently detected surrounding *bla*_*CTX*-*M*_ [16, 17].

Thus, this study intends to inquire into the prevalent trend of *bla*_*CTX*-*M*-*55*_ genes and their transferability and genetic contexts among clinical strains in Henan Province in central China.

## Methods

### Bacterial isolates, antimicrobial susceptibility testing and ESBLs confirmation

Total number of 227 unduplicated ESBL-positive *Enterobacteriaceae* [*Escherichia coli* (n = 93), *Klebsiella pneumoniae* (n = 86), *Enterobacter cloacae* (n = 13), *Enterobacter aerogenes* (n = 6), *Proteus mirabilis* (n = 7), *Citrobacter freundii* (n = 13), *Morganella morganii* (n = 3), *Serratia marcescens* (n = 5), and *Shigella flexneri* (n = 1)] clinical isolates were obtained from the First Affiliated Hospital of Zhengzhou University in Central China between September 2015 and March 2016. All strains were confirmed by using Vitek 2 (bioMérieux, France). Antimicrobial susceptibility for the *bla*_CTX-M-55_-producing strains and transconjugants were determined using Vitek 2, followed by the measurement of minimum inhibitory concentrations (MICs) utilizing the broth microdilution method (for piperacillin–tazobactam, ampicillin–sulbactam, cefotaxime, ceftazidime, cefotetan, cefepime, imipenem, ertapenem, amikacin, gentamicin, levofloxacin, and ciprofloxacin). Microbroth and agar dilution methods were standardized following the protocols from the Clinical and Laboratory Standards Institute (CLSI) [18]. The MIC results were judged by 2014 CLSI criteria [18]. All isolates were confirmed to have the ESBL phenotype through the CLSI disc confirmatory test [18]. *K. pneumoniae* ATCC 700603 and *E. coli* ATCC 25922 were used as quality control strains.

### Identification of resistance genes and the genetic contexts of *bla*_*CTX*-*M*-*55*_

To verify the emergence of plasmid-mediated ESBL genes, all ESBL-positive strains were further characterized, and plasmid DNA was extracted utilizing a Tiangen Plasmid Purification Mini Kit (Tiangen Biotech, China) referring to the protocol of manufacturer. The primer sequences presented in Table 1 were used for the *bla*_*TEM*_, *bla*_*SHV*_, and *bla*_*CTX*-*M*-*1*-*groups*_ to determine the genetic context of *bla*_*CTX*-*M*-*55*_. Purified PCR productions were sequenced immediately from two ends and compared with genes in GenBank (http://www.ncbi.nlm.nih.gov/genebank/).

**Table 1:** PCR primers characteristics in this study

PCR target	Primer name	Primer sequence (5′→3′)	Annealing temperature (°C)	Product (bp)	Reference
Upstream flanking region of *bla*_*CTX*-*M*-*55*_	*ISEcp1*-F	CAAAATGATCCCCTCGTCAAC	55	Variable	[29]
	*IS26*-F	TTACATTTCAAAAACTCTGCTTACC	57	Variable	[32]
	*bla*_*CTX*-*M*-*1*_-R	ACTTTACTGGTACTGCACAT			
Downstream flanking region of *bla*_*CTX*-*M*-*55*_	*bla*_*CTX*-*M*-*1*_-F	TTCTGGTGACRTACTTRACCCA			
	*IS903*-R	GTTTAATGACCAGCACAGT	55	364	This study
	*ORF477*-R	TCGTTTCGTGGTGCTGAATTT	57	Variable	[29]
*bla*_*CTX*-*M*-*1*_	*bla*_*CTX*-*M*-*1*_-F	CAGCGCTTTTGCCGTCTAAG	52	946	This study
	*bla*_*CTX*-*M*-*1*_-R	GGCCCATGGTTAAAAAATCACTGC			
*bla*_*TEM*_	*bla*_*TEM*_ -F	CATTTCCGTGTCGCCCTTATTC	56	800	[11]
	*bla*_*TEM*_ -R	CGTTCATCCATAGTTGCCTGAC			
*bla*_*SHV*_	*bla*_*SHV*_ -F	AGCCGCTTGAGCAAATTAAAC	55	713	[11]
	*bla*_*SHV*_ -R	ATCCCGCAGATAAATCACCAC			

### Multilocus sequence typing (MLST)

MLST for clinical *E. coli* and *K. pneumoniae* strains were detected basis on the assay discussed above [19, 20]. The sequence types (STs) and allelic profiles were assigned after comparing them to an online database (http://bigsdb.Pasteur.fr/ecoli/ecoli.html and http://bigsdb.Pasteur.fr/klebsiella/klebsiella.html).

### Conjugation experiments

Conjugative assays were performed using the methods discussed above [7]. The *bla*_*CTX*-*M*-*55*_-positive isolates served as donors, and *E. coli* C600 functioned as a recipient. Transconjugants were screened on Mueller–Hinton agar containing 750 μg/ml rifampin and 100 μg/ml ampicillin. The existence of *bla*_*CTX*-*M*-*55*_ in the transconjugants was identified through antimicrobial susceptibility, PCR and DNA sequencing.

## Results

### Identification of *bla*_*CTX*-*M*-*55*_-positive isolates and their antimicrobial susceptibility and resistance determinants

Based on the results of this study, among 227 ESBL-positive *Enterobacteriaceae,* 13 [13/227 (5.73%)] were identified as *bla*_*CTX*-*M*-*55*_-positive, including 7/93 *E. coli*, 3/86 *K. pneumoniae*, 1/13 *C. freundii*, 1/3 *M. morganii*, and 1/5 *S. marcescens*, which were collected from blood (n = 6), urine (n = 3), and sputum (n = 3) samples (Table 2). The antimicrobial susceptibility analyses of the 13 *bla*_*CTX*-*M*-*55*_-positive isolates are presented in Table 3. All strains were insusceptible to third-generation cephalosporins (ceftazidime and cefotaxime), fluoroquinolones (levofloxacin and ciprofloxacin), and gentamicin. In addition, 100% susceptibility to amikacin was found. The isolates were also generally sensitive to imipenem (10/13, 76.92%) and ertapenem (9/13, 69.23%), whereas all the other microbiotics, including cefepime, cefotetan and piperacillin–tazobactam, exhibited moderate to low susceptibility. Additionally, among 13 isolates carrying *bla*_*CTX*-*M*-*55*_, 5 isolates contained *bla*_*TEM*_, and 2 isolates had both *bla*_*TEM*_ and *bla*_*SHV*_ (Table 3).

**Table 2:** Characterisitics of blaCTX-M-55-positive isolates

Isolate	Specimen	Department	ESBL	MLST
EC30	Blood	Urology	TEM/SHV	ST156
EC32	Blood	Gastroenterology	–	ST305
EC44	Urine	Respiration	–	ST182
EC45	Sputum	ICU	TEM	ST305
EC52	Blood	Urology	–	ST381
EC54	Blood	EICU	–	ST446
EC67	Blood	Gastroenterology	TEM	ST2
KP26	Sputum	Thoracic surgery	TEM	ST148
KP37	Blood	General surgery	TEM/SHV	ST269
KP146	Urine	Urology	TEM	ST37
CF547	Urine	Urology	–	–
MM556	Drainage fluid	Anus and intestine surgery	TEM	–
SM554	Sputum	Neurosurgery	–	–

EC, *E. coil*; KP, *K. pneumoniae*; CF, *C. freundii*; MM, *M. morganii*; SM, *S. marcescens*; ICU, intensive care unit; EICU, emergency ICU

**Table 3:** Antibiotic susceptibilities of *bla*_*CTX*-*M*-*55*_-positive and their transconjugants

Isolate	Antibiotic^a^ susceptibility (μg/ml)											
	SAM	TZP	CTX	CAZ	CTT	FEP	IPM	ETP	AMK	GEN	LVX	CIP
EC30	> 256	> 256	> 256	> 256	> 256	> 256	< 1	2	< 2	64	> 32	> 32
EC32	> 256	8	> 256	> 256	8	> 256	< 1	1	< 2	64	16	8
EC44	> 256	8	> 256	> 256	8	> 256	< 1	<0.5	< 2	32	16	8
EC45	> 256	64	> 256	> 256	32	> 256	< 1	<0.5	< 2	32	16	8
EC52	> 256	8	> 256	> 256	8	2	< 1	<0.5	< 2	64	> 32	8
EC54	> 256	8	> 256	> 256	8	16	< 1	<0.5	< 2	32	16	8
EC67	> 256	> 256	> 256	> 256	>256	> 256	< 1	2	< 2	32	> 32	8
KP26	> 256	64	> 256	> 256	32	> 256	< 1	1	< 2	32	> 32	8
KP37	> 256	> 256	> 256	> 256	> 256	> 256	8	2	< 2	32	16	8
KP146	> 256	64	> 256	> 256	> 256	> 256	8	2	< 2	64	> 32	8
CF547	> 256	8	> 256	64	8	> 256	< 1	< 0.5	< 2	64	16	8
MM556	> 256	64	> 256	> 256	> 256	2	< 1	< 0.5	< 2	32	16	8
SM554	> 256	8	> 256	64	8	> 256	8	< 0.5	4	32	16	8
*E. coil* transconjugants												
EC30-C600	> 256	64	> 256	> 256	> 256	> 256	< 1	2	< 2	64	< 0.25	< 0.25
EC32-C600	> 256	8	> 256	> 256	8	8	< 1	1	< 2	32	< 0.25	< 0.25
EC44-C600	> 256	8	128	64	4	4	< 1	< 0.5	< 2	32	< 0.25	< 0.25
EC45-C600	> 256	32	> 256	> 256	32	16	< 1	< 0.5	< 2	16	< 0.25	< 0.25
EC52-C600	> 256	8	> 256	128	8	2	< 1	< 0.5	< 2	32	< 0.25	< 0.25
EC54-C600	> 256	8	128	> 256	8	16	< 1	< 0.5	< 2	32	< 0.25	< 0.25
EC67-C600	> 256	> 256	> 256	128	> 256	> 256	< 1	2	< 2	16	< 0.25	< 0.25
KP26-C600	> 256	32	> 256	> 256	16	8	< 1	1	< 2	32	< 0.25	< 0.25
KP37-C600	> 256	64	64	128	> 256	8	8	2	< 2	16	< 0.25	< 0.25
KP146-C600	> 256	32	> 256	> 256	> 256	> 256	8	2	< 2	64	< 0.25	< 0.25
CF547-C600	> 256	4	> 256	64	8	32	< 1	< 0.5	< 2	32	< 0.25	< 0.25
MM556-C600	> 256	64	64	> 256	> 256	2	< 1	< 0.5	< 2	16	< 0.25	< 0.25
SM554-C600	> 256	8	> 256	64	8	32	8	< 0.5	4	32	< 0.25	< 0.25
EC-C600	< 2	< 4	< 1	< 1	< 4	< 1	< 1	< 0.5	< 2	< 1	< 0.25	< 0.25

EC, *E. coil*; KP, *K. pneumoniae*; CF, *C. freundii*; MM, *M. morganii*; SM, *S. marcescens*

^a^SAM, ampicillin–sulbactam (1/0.5–256/128) [(μg/ml) for each agent, and the numbers in parentheses indicate the test range]; TZP, piperacillin–tazobactam (0.5/4–256/4); CTX, cefotaxime (0.03–256); CAZ, ceftazidime (0.03–256); CTT, cefotetan (0.03–256); FEP, cefepime (0.015–256); IPM, imipenem (0.06–32); ETP, ertapenem (0.004–32); AMK, amikacin (0.5–256); GEN, gentamicin (0.25–256); LVX, levofloxacin (0.008–32); CIP, ciprofloxacin (0.004–32)

### MLST and conjugal transfer of the *bla*_*CTX*-*M*-*55*_ gene

MLST was detected for *bla*_*CTX*-*M*-*55*_-positive *E. coli* and *K. pneumoniae* strains. Nine types of MLST were detected among the 7 *E. coli* strains (ST156, ST305, ST182, ST381, ST446 and ST2) and 3 *K. pneumoniae* strains (ST148, ST269 and ST37). Two *E. coli* isolates (EC32 and EC45) shared the same ST type (ST305) (Table 2). Conjugative assays indicated that all *bla*_*CTX*-*M*-*55*_ plasmids were transmitted to *E. coli* C600 from 13 donors successfully through conjugation. Although all transconjugants exhibited resistance to cefotaxime and ceftazidime, they were all sensitive to fluoroquinolones. Additionally, the *bla*_*TEM*_ and *bla*_*SHV*_ resistance genes were transformed to *E. coli* C600 with the *bla*_*CTX*-*M*-*55*_ for some isolates (Table 3).

### Genetic contexts of *bla*_*CTX*-*M*-*55*_

**Fig. 1:**
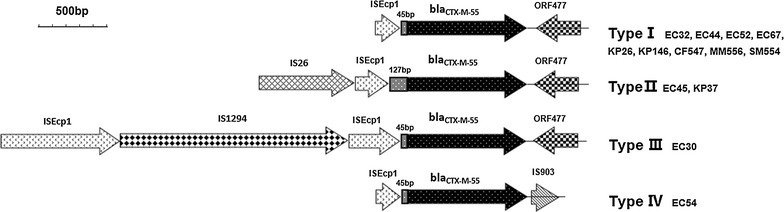
Surrounding the regions of *bla*_*CTX*-*M*-*55*_ gene in this study. Type I architecture (*ISEcp1∆*-*bla*_*CTX*-*M*-*55*_-*∆ORF477*) was found in isolates (EC32, EC44, EC52, EC67, KP26, KP146, CF547, MM556, SM554) (GenBank Accession Numbers: KX889071; KX889081; KX889072; KX889073; KX889074; KX889075; KX889076; KX889077; KX889078); Type II architecture (*IS26*-*∆ISEcp1*-*bla*_*CTX*-*M*-*55*_-*∆ORF477*) was found in isolates (EC45, KP37) (GenBank Accession Numbers: KX889079; KX889080); Type III architecture (*ISEcp1∆*-*IS1294*-*∆ISEcp1*-*bla*_*CTX*-*M*-*55*_-*∆ORF477*) was found in isolate (EC30) (GenBank Accession Number: KX889070); Type IV architecture (*ISEcp1∆*-*bla*_*CTX*-*M*-*55*_-*∆IS903*) was found in isolate (EC54) (GenBank Accession Numbers: KX898438 and KX898439)

The flanking region of *bla*_*CTX*-*M*-*55*_ is presented in Fig. 1. Four different architectures [type I (9 isolates), type II (2 isolates), type III (1 isolate), and type IV (1 isolate)] were identified regarding the genetic contexts of the plasmid-mediated *bla*_*CTX*-*M*-*55*_ genes. Type I architecture (*ISEcp1∆*-*bla*_*CTX*-*M*-*55*_-*∆ORF477*) was the most common and was identified in 9 (69.23%) of 13 *bla*_*CTX*-*M*-*55*_-positive isolates; the occurrence of type II (*IS26*-*∆ISEcp1*-*bla*_*CTX*-*M*-*55*_-*∆ORF477*) and type III architecture (*ISEcp1∆*-*IS1294*-*∆ISEcp1*-*bla*_*CTX*-*M*-*55*_-*∆ORF477*) was similar to type I architecture, although *ISEcp1* was disrupted by *IS26* in type II and by *IS1294* in type III. Type IV (*ISEcp1∆*-*bla*_*CTX*-*M*-*55*_-*∆IS903*) was characterized by the existence of *IS903*, which was detected firstly downstream of *bla*_*CTX*-*M*-*55*_.

## Discussion

Since the CTX-M-55 firstly reported in 2006, it has been identified in *E. coli, K. pneumoniae, S. flexneri* and *Salmonella enteritidis* [3, 7, 10]. For all we know, *bla*_*CTX*-*M*-*55*_ in *C. freundii* and *M. morganii* is firstly detected in this study. In addition, 13/227 isolates were identified as *bla*_*CTX*-*M*-*55*_-positive in just 7 months. This rate far surpasses other ESBLs [21–23], which demonstrates the rapid dissemination of *bla*_*CTX*-*M*-*55*_. Notably, all *bla*_*CTX*-*M*-*55*_-positive isolates were identified as multiple drug-resistant (MDR) bacteria that are strongly resistant to ceftazidime and cefotaxime (MIC > 256 μg/ml). More significantly, molecular characterization also revealed that most of the *bla*_*CTX*-*M*-*55*_-positive isolates harbored *bla*_*TEM*_. In addition, some isolates contained *bla*_*SHV*_. These results imply that the spreading of *bla*_*CTX*-*M*-*55*_ over many different genera of *Enterobacteriaceae* is activated in hospitals in Henan Province, which represents a public health issue due to the inability to treat these bacteria.

Two *E. coli* isolates (EC32 and EC45) isolated from two different departments (Gastroenterology and ICU) shared the same ST type (ST305), which suggests that they are clonally related. However, the data indicate that the CTX-M-55-positive *E. coli* and *K. pneumoniae* strains identified in our study were not clonally related by MLST, which indicates that there is no specific ST in Henan Province. This finding contrasts with observations in the region of European and North American, where a high prevalence of ST131 has been observed [24]. Furthermore, this study demonstrates the association of eight STs [ST305, ST182, ST381, ST446 and ST2 (*E. coli*) and ST148, ST269 and ST37 (*K. pneumoniae*)] with the products of CTX-M-55 first time, which means *bla*_*CTX*-*M*-*55*_ has been actively spreading among *Enterobacteriaceae* in China. Given our focus on conjugative assays, the 13 transconjugants all exhibited resistance to cefotaxime and ceftazidime but sensitivity to fluoroquinolones, which was consistent with the original isolates. These results suggest that the plasmid-mediated *bla*_*CTX*-*M*-*55*-_gene is to answer for an ESBL phenotype with poor susceptibility to cefotaxime and ceftazidime and exhibits a strong transferability of resistance. This finding also indicates that fluoroquinolones should be used for the therapy of *bla*_*CTX*-*M*-*55*_-positive pathogen infections in clinical settings. Interestingly, our data indicate that some original isolates were resistant to cefepime, but the transconjugants were susceptible, which suggests that the original isolates may include other resistance genes that promote resistance to cefepime. We did not detect these genes in our study. These resistance genes cannot be transmitted through conjugative assays and are not located on the chromosome. Thus, this mechanism requires further study.

The sporadic existence of CTX-M-55-positive strains in mainland China has been occasionally detected. In some surveys, CTX-M-55 incidence has surpassed that of CTX-M-15 [25]. Heterogeneous genetic contexts may indicate the dissemination and mobilization of *bla*_*CTX*-*M*-*55*_. As shown in Fig. 1, all isolates were detected *ISEcp1*, locating upstream of *bla*_*CTX*-*M*-*55*_; this region contain the promoter sequence (− 35 and − 10) and act as a significant role in the expression and mobilization of the β-lactamase genes [12, 15, 26]. Moreover, the presence of *ISEcp1* in this cross-species study indicates that the complete or partial insertion sequence was probably excised along with CTX-M-55 during horizontal transfer. Previous reports demonstrated that the disruption of the *ISEcp1* element by *IS26* was linked to the promotion of *bla*_*CTX*_ gene dissemination [27, 28]. Interestingly, as previously reported, *ISEcp1* disruption by *IS1294* in *bla*_*CTX*-*M*-*55*_ was detected from a chicken in China, which may contribute to the mobilization of bla_CTX-M-55_ [29]. Remarkably, the two *E. coli* strains [EC30 (this study) and *E. coli* C21 [29] ] shared the same MLST type (ST156), which suggests that these isolates are clonally related. This coincidence implies that *bla*_*CTX*-*M*-*55*_ is likely to be transferred from animals to the clinical setting. Fey et al. found that a 12-year-old boy acquired ceftriaxone-resistant *Salmonella enterica* serotype Typhimurium from cattle [30]. Jing Zhang et al. reported that CTX-M-55 had already been transmitted to humankind from animals and is distributed among both hospitals and community in China. The findings of our investigation and previous studies indicate that *bla*_*CTX*-*M*-*55*_ can be transmitted to humankind from food and can enhance clinical resistance. Notably, the novel arrangement *ISEcp1∆*-*bla*_*CTX*-*M*-*55*_-*∆IS903* is characterized by the element of *IS903* which is detected downstream of *bla*_*CTX*-*M*-*55*_ first time and often identified by the context of other *bla*_*CTX*-*M*_ genes [31]. The mechanism responsible for its presence remains unclear. According to Poirel et al., *ISEcp1*, *bla*_*CTX*_ and *IS903* form a putative transposon, and this block of genes could be disseminated by transposition [26, 32]. This finding implies that *IS903* contributes to the dissemination of *bla*_*CTX*-*M*-*55*_, which requires further study. Therefore, our findings strongly suggest that genetic elements (*ISEcp1*, *ORF477*, *IS26*, *IS1294*, and *IS903*) are involved in the inter-species and intra-species mobilization and dissemination of *bla*_*CTX*-*M*-*55*_. Additionally, CTX-M-55-harboring isolates in animals may act as a potential storage of bacterial that is spread in clinical.

## Conclusions

This investigation reminds a high occurrence rate of CTX-M-55-producing ESBLs in patients from different departments at the First Affiliated Hospital of Zhengzhou University in Henan Province. These plasmid-mediated *bla*_*CTX*-*M*-*55*_-positive isolates are contributed to the transmission of *bla*_*CTX*-*M*-*55*_ to new species and new hosts by conjugation. Data obtained in this study suggest that the genetic context of *bla*_*CTX*-*M*-*55*_, especially *ISEcp1*, act as a vital part in the mobilization, dissemination and expression of drug resistance determinants. We also demonstrated a novel arrangement of *bla*_*CTX*-*M*-*55*_ (*ISEcp1∆*-*bla*_*CTX*-*M*-*55*_-*∆IS903*). Thus, the presence of MDR *Enterobacteriaceae* contains conjugative plasmids that co-harbor other IS elements, such as *ISEcp1*, should be surveilled worldwide because the active transfer and high prevalence of these pathogenic will significantly decrease our further selection of clinical therapies. Further studies on this issue should be performed to help us obtain a deeper understanding of the transmission and dissemination of plasmid-mediated *bla*_CTX-M-55_ in different genetic platforms.

## Nucleotide sequence accession number

The nucleotide sequences presence in this study have been submitted to GenBank under the following accession numbers: KX889070 (*E. coli*: EC30); KX889071 (*E. coli*: EC32); KX889072 (*E. coli*: EC52); KX898438 and KX898439 (*E. coli*: EC54); KX889073 (*E. coli*: EC67); KX889074 (*K. pneumoniae*: KP26); KX889075 (*K. pneumoniae*: KP146); KX889076 (*C. freundii*: CF547); KX889077 (*M. morganii*: MM556); KX889078 (*S. marcescens*: SM554); KX889079 (*E. coli*: EC45); KX889080 (*K. pneumoniae*: KP37); KX889081 (*E. coli*: EC44).
